# First Results from a Screening of 300 Naturally Occurring Compounds: 4,6-dibromo-2-(2′,4′-dibromophenoxy)phenol, 4,5,6-tribromo-2-(2′,4′-dibromophenoxy)phenol, and 5-epi-nakijinone Q as Substances with the Potential for Anticancer Therapy

**DOI:** 10.3390/md17090521

**Published:** 2019-09-05

**Authors:** Saskia Mayer, Marie Prechtl, Pia Liebfried, Ron-Patrick Cadeddu, Fabian Stuhldreier, Matthias Kohl, Folker Wenzel, Björn Stork, Sebastian Wesselborg, Peter Proksch, Ulrich Germing, Rainer Haas, Paul Jäger

**Affiliations:** 1 Faculty Medical and Life Sciences, Campus Villingen-Schwenningen, Hochschule Furtwangen University, 78120 Furtwangen, Germany (S.M.) (M.P.) (M.K.) (F.W.); 2 Department of Hematology, Oncology and Clinical Immunology, University Hospital Düsseldorf, 40225 Düsseldorf, Germany (P.L.) (R.-P.C.) (U.G.) (R.H.); 3 Institute for Molecular Medicine I, University Hospital Düsseldorf, 40225 Düsseldorf, Germany (F.S.) (B.S.) (S.W.); 4 Institute of Pharmaceutical Biology and Biotechnology, Heinrich Heine University, 40225 Düsseldorf, Germany

**Keywords:** marine sponge derived natural products, polybrominated diphenyl ethers, sesquiterpene aminoquinone, bioactive natural products, cytotoxic activity, apoptosis, peripheral blood mononuclear cells, primary leukemic cells, acute myeloid leukemia, drug leads

## Abstract

There is a variety of antineoplastic drugs that are based on natural compounds from ecological niches with high evolutionary pressure. We used two cell lines (Jurkat J16 and Ramos) in a screening to assess 300 different naturally occurring compounds with regard to their antineoplastic activity. The results of the compounds 4,6-dibromo-2-(2′,4′-dibromophenoxy)phenol (P01F03), 4,5,6-tribromo-2-(2′,4′-dibromophenoxy)phenol (P01F08), and 5-epi-nakijinone Q (P03F03) prompted us to perform further research. Using viability and apoptosis assays on the cell lines of primary human leukemic and normal hematopoietic cells, we found that P01F08 induced apoptosis in the cell lines at IC50 values between 1.61 and 2.95 μM after 72 h. IC50 values of peripheral blood mononuclear cells (PBMNCs) from healthy donors were higher, demonstrating that the cytotoxicity in the cell lines reached 50%, while normal PBMNCs were hardly affected. The colony-forming unit assay showed that the hematopoietic progenitor cells were not significantly affected in their growth by P01F08 at a concentration of 3 μM. P01F08 showed a 3.2-fold lower IC50 value in primary leukemic cells [acute myeloid leukemia (AML)] compared to the PBMNC of healthy donors. We could confirm the antineoplastic effect of 5-epi-nakijinone Q (P03F03) on the cell lines via the induction of apoptosis but noted a similarly strong cytotoxic effect on normal PBMNCs.

## 1. Introduction

According to recent estimates, more than 8.9 million people die of cancer worldwide every year [1]. Independent of the type of cancer, our general understanding of the processes underlying cancer is that the malignant transformation within the cell of origin is accompanied by multiple genetic alterations and mutations. The latter are caused by a variety of environmental and lifestyle factors such as smoking or nutrition habits, viruses, and aging, which are generally associated with increasing genetic instability [2]. The systemic treatment of patients with cancer still largely relies on cytostatic agents also affecting normal cells to some extent. As a result, side effects are encountered, causing morbidity with substantial impact on the quality of life [3]. Cytotoxic drugs are often modified compounds derived from natural products. For example, Vinca alkaloids represent compounds from the Madagascar periwinkle plant [4], while Taxol is retrieved from the bark of the western yew [5]. 

With a better understanding of the molecular mechanisms underlying cancer, new types of targeted therapies have emerged. Nowadays, targeted therapies based on molecularly designed small molecules are on the rise. For instance, the tyrosine kinase inhibitor Imatinib was developed to target the fusion gene BCR-ABL, which is constitutively activated in chronic myeloid leukemia (CML). This kind of molecular precision therapy was extremely successful, providing long term hematological and molecular remissions for patients with CML without significant toxicity [6]. An example for a targeted therapy rooted in a natural compound is Midostaurin. It is a semi-synthetic derivative of the structurally related Staurosporine, which was first isolated from the soil-derived actinobacteria *Streptomyces staurosporeus*. Midostaurin resulted from a drug discovery effort to improve the protein kinase C inhibitory activity of Staurosporine [7] and targets constitutively activated mutant FMS-like tyrosine kinase-3 (FLT3), which is expressed in a subpopulation of patients with acute myeloid leukemia (AML) [8]. The search for new naturally occurring compounds with potential antineoplastic activity starts in ecological niches with high evolutionary pressure. For example, bioactive metabolites of marine organisms are particularly efficient in their protection, adaptation, and survival within the specific ecosystem of the sea. Sponges mainly rely on chemical defense mechanisms, allowing successful competition for space and resources [9]. Interestingly, marine compounds interfere with signaling pathways relevant for malignant cells such as those involved in cell death and inflammatory processes [10]. These organisms therefore represent a rich but sparsely exploited source of compounds with a high degree of structural and anti-neoplastic properties [11].

**Figure 1:**
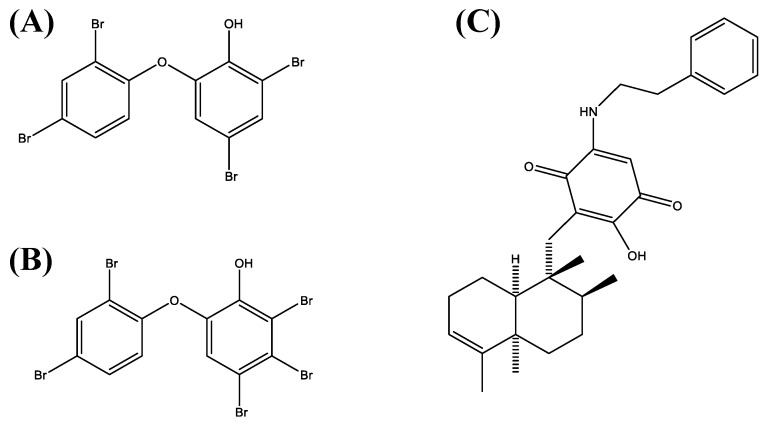
Structural formula of (**A**) 4,6-dibromo-2-(2′,4′-dibromo-phenoxy)phenol (P01F03), (**B**) 4,5,6-tribromo-2-(2′,4′-dibromophenoxy)phenol (P01F08), and (**C**) 5-epi-Nakijinone Q (P03F03) (all provided by the Institute of Pharmaceutical Biology and Biotechnology, Heinrich Heine University, Düsseldorf, Germany).

Based on the results of a screening of 300 natural compounds (P01-P05, B-G, 02-11) contained within the biobank of the Institute of Pharmaceutical Biology and Biotechnology at the Heinrich Heine University Düsseldorf on the cell lines Jurkat (T cell leukemia) and Ramos (B cell lymphoma), we chose three different compounds, i.e., 4,6-dibromo-2-(2′,4′-dibromophenoxy)phenol (P01F03), 4,5,6-tribromo-2-(2′,4′-dibromophenoxy)phenol (P01F08), and 5-epi-nakijinone Q (P03F03). They were selected because they showed strong antiproliferative activity on cell lines and are somewhat incompletely characterized in the literature. The polybrominated diphenyl ether derivatives P01F03 (Figure 1A) and P01F08 (Figure 1B) were isolated from the marine sponge *Dysidea* sp. [12], while P03F03 (Figure 1C) was derived from the sponge *Dactylospongia metachromia* [13]. Going beyond the cell line models, we further investigated these compounds using primary malignant cells obtained from patients with myeloid stem cell disorder. With the perspective to use them for the treatment of patients with leukemia, it was mandatory to assess the natural compounds with regard to their potential cytotoxic side effects on healthy human peripheral blood mononuclear cells using suspension culture and colony-forming unit assays. Our results demonstrated sufficient antineoplastic activity for all three natural compounds without undue toxicity of the two polybrominated diphenyls as far as the normal blood and the progenitor cells are concerned. Further studies concentrating on other types of human leukemia and more elaborate assays such as long-term culture initiating cell assays for determining hematopoietic toxicity may help to pave the way towards clinical application.

## 2. Methods

### 2.1. Compounds

P01F03, P01F08, and P03F03 were obtained from the compound library of the Institute for Pharmaceutical Biology and Biotechnology of the Heinrich Heine University Düsseldorf freshly prepared and dissolved in DMSO. Until use for the assays, they were kept at −20 °C in a temperature-controlled refrigerator. 

### 2.2. Cell Line Culturing Conditions

Jurkat J16 (acute T cell leukemia cells), Ramos (Burkitt’s lymphoma B lymphocytes), HL-60 (acute promyelocytic leukemia cells), and THP-1 (acute monocytic leukemia cells) (each 2 × 10^6^ cells plated) were cultivated in T75 cm^2^ culture flasks in RPMI-1640 medium supplemented with 10% fetal bovine serum (FBS) and 1% penicillin/streptomycin/L-glutamine (PSG) (all from Sigma-Aldrich Chemie GmbH, Taufkirchen, Germany) in a humidified atmosphere of 5% CO_2_ at 37 °C. In accordance with the manufacturer’s recommendation, no growth factors were added to the culture medium of cell lines (all purchased from DMSZ, Braunschweig, Germany). The cell density was determined using a Neubauer chamber slide. 

### 2.3. Patients’ Samples and Healthy Controls

In this work, peripheral blood samples (PB) from a total of 6 patients (median age: 67 years, range: 40–74 years) covering two AML common subtypes according to the World Health Organization (WHO) classification with a median peripheral blast count of 54% (range: 18–92%) were included. Samples were obtained at diagnosis at the Department of Hematology, Oncology and Clinical Immunology, of the University Hospital Düsseldorf. Patients’ characteristics with hematological parameters are given in Table 1. Control samples were derived from 9 healthy individuals undergoing apheresis of enriched peripheral blood mononuclear cells as donors for allogenic stem cell transplantation. The aliquots were provided from the Institute of Transplantation Diagnostics and Cell Therapeutics of the University Hospital Düsseldorf. The study was approved by the local ethical review committee (study number: 5944R; registration ID: 2017044215), and all patients gave written informed consent.

### 2.4. Isolation and Cultivation of Mononuclear Cells and CD34+ Cells

Mononuclear cells were obtained from peripheral blood (PB) or apheresis products (AP) following density gradient separation. An interim step lysis of remaining erythrocytes with hypotonic 0.83% ammonium chloride was performed. CD34+ cells were positively selected from PB or AP-derived mononuclear cells (MNC) by immunomagnetic cell separation (Miltenyi Biotec, Bergisch-Gladbach, Germany) as published (Schroeder 2009). 

The cells were cultivated with a cell density of 100–1000 cells/µL in T75 cm^2^ culture flasks in RPMI-1640 medium supplemented with 10% FBS and 1% PSG in a humidified atmosphere of 5% CO_2_ at 37 °C. The cell density was determined using a Neubauer chamber slide. To sufficiently maintain healthy and patient MNCs and CD34+ cells in culture, the RPMI medium contained Interleukin 3 (IL-3), IL-6, stem cell factor (SCF), and FLT3-ligand (all 10 ng/mL, PreproTech GmbH, Hamburg, Germany). 

### 2.5. 3-(4,5-dimethylthiazol-2-yl)-2,5-diphenyltetrazolium bromide (MTT) Assay

Cell viability was determined by the MTT assay. It is a colorimetric method based on the ability of cells to convert the yellow MTT 3-(4,5-dimethylthiazol-2-yl)-2,5-diphenyltetrazolium bromide substrate into a blue formazan product. This reduction is NAD(P)H-dependent and, as a result, the absorbance of the purple dye is proportional to the number of viable cells. 

For cell lines, 50 µL of cell suspension was added in each well of a 96-well plate. The cell density was 1 × 10^6^ cells/mL for an incubation time of 24 h and 0.25 × 10^6^ cells/mL for an incubation time of 72 h. Since primary cells of patients with a hematological neoplasia have a lower proliferation rate than cell lines, 125 µL of cell suspension were used with a cell density of 6 × 10^5^ cells/mL for an incubation time of 72 h. The two-fold concentrated compound-dilution was added (cell lines: 50 µL, MNC: 125 µL) so that the final volume in each well amounted to 100 μL/250 µL. All samples were tested in triplicates. DMSO (0.1% *v*/*v*), staurosporine (STS, 10 µM) (both diluted in RPMI medium), and medium were used as controls. After a 24 or 72 h incubation in a humidified atmosphere of 5% CO_2_ at 37 °C, 20 μL MTT solution (dissolved in ddH_2_O, 5 mg/mL, sterile-filtered) were added and incubated for another hour. MNCs of patient samples had a lower metabolic rate. These samples, therefore, were incubated for two hours with MTT solution. The cells were then centrifuged at 600× *g* and 4 °C for 5 min, and the supernatant was removed. DMSO (cell lines: 100 μL; MNCs: 70–90 μL) was added, and the cells were incubated in the dark at room temperature for 20–30 min on a rocking platform. When the crystals dissolved, the samples were transferred to a 96-well plate with a flat bottom, and the absorbance was measured in a plate reader using a wavelength of 570 nm and a reference wavelength of 650 nm. The mean values of the staurosporine controls were subtracted from all other values, since a relative viability of 0% could be assumed here. The relative viability of the cells incubated with DMSO was set to 100%. For the remaining absorption values, a relative viability was determined. These relative viability values were calculated with the software Prism via a non-linear regression analysis, and the IC50 value was calculated. The mean inhibitory concentration (IC50) is the concentration of an inhibitor at which half-maximum inhibition is observed. All IC50 values are given with the 95% confidence interval.

### 2.6. Propidium Iodide (PI) Uptake

The propidium iodide (PI) uptake assay is a method for detecting cell death. Therefore, the plasma membrane integrity is determined via the uptake of propidium iodide (PI). It is a small fluorescent molecule that binds to DNA. It cannot passively pass into cells that have an intact plasma membrane, as is the case for viable or early apoptotic cells. As a result, only a defective plasma membrane allows the uptake of PI. The method does not differentiate between late apoptotic and necrotic cells, as the plasma membranes become permeable in both scenarios. Accordingly, every sample was additionally incubated with the broad-range caspase inhibitor Q-VD-OPh (QVD; 1:1000), which inhibits apoptosis. With the assemblage of the data of each sample with and without QVD, apoptosis and necrosis could be distinguished.

Subsequently, 50 μL cell suspensions were added in each well of a 96-well plate. The cell density was 1 × 10^6^ cells/mL for incubation times of 24 h and 72 h. Then, 50 μL of the two-fold concentrated compound dilution was added so that the final concentration per compound was 10 µM and the volume in each well amounted to 100 μL. All samples were tested in triplicates. After the incubation time, the plate was centrifuged at 800× *g* and 4 °C for 5 min. After, the supernatant was removed and 150 μL of PI solution (0.01 mg/mL in PBS) was added. Subsequently, the plate was incubated in the dark at RT for 15 min. After that, every sample (10,000 cells) was measured by flow cytometry.

### 2.7. DEVDase Assay for the Fluorometric Analysis of Caspase-3-Activity

This assay serves for determination of the apoptosis rate. Caspase-3 is activated in the course of apoptosis. To determine the apoptosis rate, the synthetic fluorophore-tagged substrate of caspase-3 (Ac-DEVD-AMC) is added. It is cleaved by activated caspase-3, which leads to the release of the fluorophore AMC (7-amido-4-methylcoumarin). As a result, the measured fluorescence is directly proportional to the DEVDase activity.

For this assay, 1 × 10^5^ cells per well were seeded in a 96-well plate. For determining kinetics of the caspase-3 activity over 8 h, every 2 h, the compound dilution was added to a part of the wells. At the end, the caspase-3 activity was measured after a treatment for 2, 4, 6, and 8 h of incubation. The final volume in each well reached 100 μL, and the samples were tested in triplicates. After 8 h of incubation, the cells were centrifuged at 800× *g* and 4 °C for 5 min, the supernatant was removed, and the cells were frozen at −80 °C. During the following steps, the cells were kept on ice. At first, the cells were thawed on ice, and 50 μL lysis buffer (20 mM HEPES, 84 mM KCl, 10 mM MgCl_2_, 200 µM EDTA, 200 µM ethylene glycol-bis(2-aminoethylether)-N,N,N´,N´-tetraacetic acid (EGTA), 0.5% nonyl phenoxypolyethoxylethanol NP-40) containing the protease inhibitors leupeptin, aprotinin, and pepstatin were added to each well. Subsequently, the cells were incubated on ice for 10 min. Then, 40 μL of the sample were transferred in a new black 96-well plate with a flat bottom, and 150 μL reaction buffer (50 mM HEPES, 100 mM NaCl, 10% Sucrose, 0.1% 3-[(3-Cholamidopropyl)dimethylammonio]-1-propanesulfonate hydrate (CHAPS), 2 mM CaCl_2_) including the substrate Ac-DEVD-AMC were added. Finally, the fluorescence was measured every 2 min for 2.5 h at 37 °C with an excitation of 360 nm and an emission of 450 nm. To evaluate this assay, the linear rise of the fluorescence was determined. The mean of the control samples “DMSO” was set as “1”.

### 2.8. Semisolid Clonogenic Assays

To assess colony-forming capacity of healthy CD34+ hematopoietic stem and progenitor cells (HSPC) after incubation with compound P01F08, colony forming unit assays (CFU) were performed. Therefore, 1 × 10^5^ MNC were incubated for 24 and 72 h on a 96-well plate with P01F08 in 4 different concentrations (0.3, 1, 3, 10 μM) and under previously mentioned cell culture conditions. Incubations with medium only, DMSO (0.1% *v*/*v*), and staurosporine (STS, 10 µM) were used as controls.

After incubation, viable cells were determined by the CASY cell counter. Then, 50,000 viable cells were seeded in semisolid ready-to-use methylcellulose growth medium (MethoCult H4436; Stem Cell Technologies, Vancouver, BC, Canada) in a 24-well plate and incubated for 14 days at 37 °C and 5% CO_2_ under humidified conditions. Subsequently, the colonies were counted and differentiated in red precursors (colony-forming unit-erythroid, CFU-E; burst-forming unit-erythroid, BFU-E), white precursors (colony-forming unit-granulocyte, -monocyte, CFU-GM; colony-forming unit-granulocyte; CFU-G; colony-forming unit-monocyte, CFU-M) and colony-forming unit-granulocyte, -erythrocyte, -monocyte, -megakaryocyte (CFU-GEMM) by light microscopy (Axiovert 25 microscope Zeiss, Jena, Germany).

### 2.9. Statistical Analyses

Statistical analyses were performed using Prism 5.01 (GraphPad Software Inc., La Jolla, CA, USA) and Microsoft Excel 2016 (Microsoft Inc., Redmont, WA, USA) with details given in the respective figure legend. Significance of MTT and DEVDase assays was determined using Welch’s t-tests of the peripheral blood mononuclear cells (PBMNCs) against the cell lines and the AML cells. For PI uptake and CFU assays, the significance between treated against untreated as well as DMSO control against treated groups was tested. The significance is indicated by asterisks in the figures (no star: *p*-value > 0.05, *: *p*-value ≤ 0.05, **: *p*-value ≤ 0.01, ***: *p*-values ≤ 0.001).

## 3. Results

### 3.1. Cytotoxicity Screening of 300 Natural Compounds

**Figure 2:**
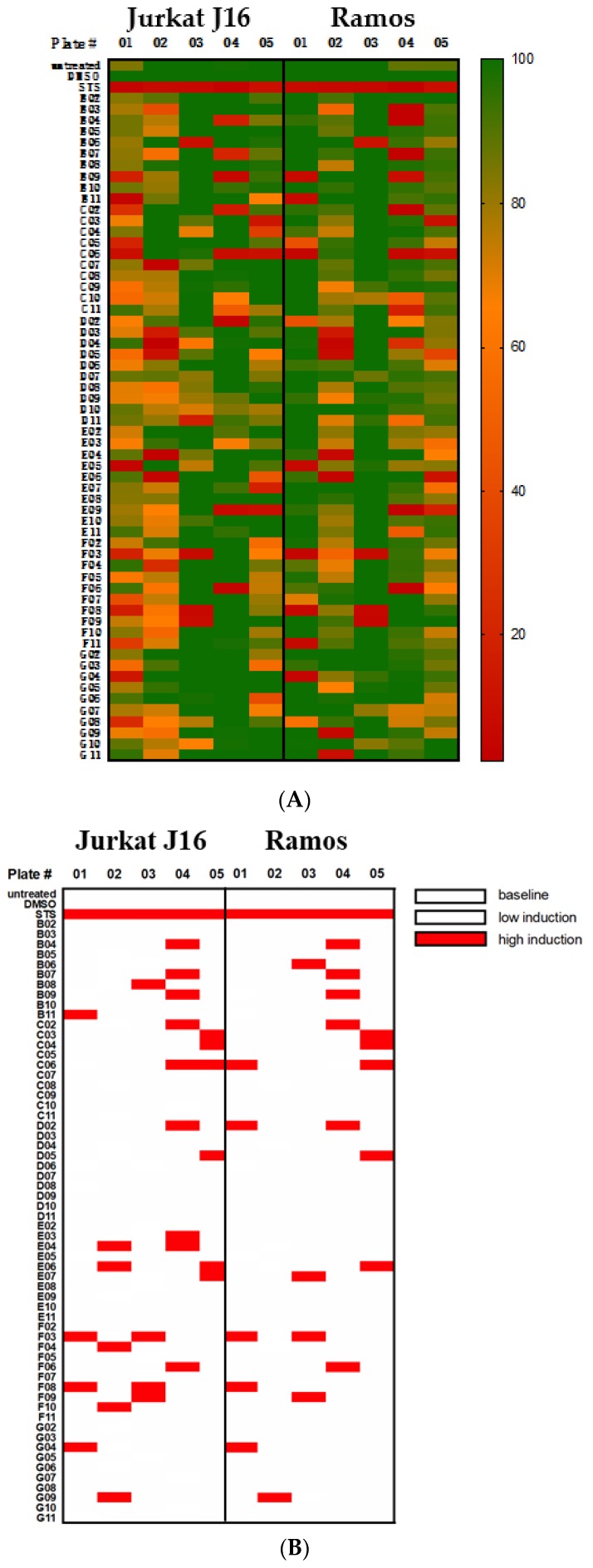

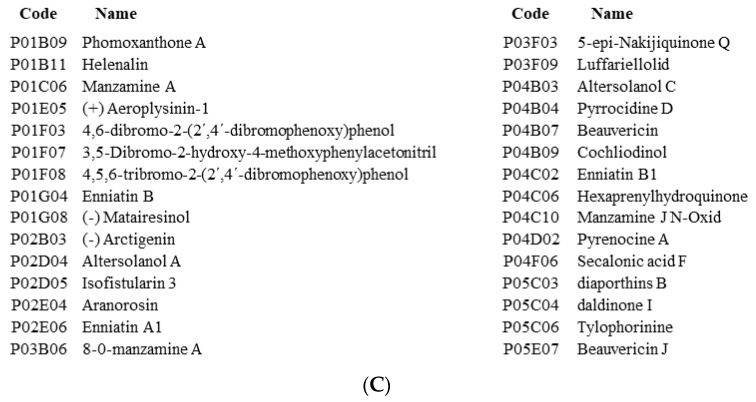
Cytotoxic effect (**A**) and apoptosis induction (**B**) of 300 natural compounds on Jurkat J16 and Ramos. Compound codes are composed of Plate # (columns P01-P05) and individual well labeling (row B02-G11). (**A**) MTT assay after 72 h of incubation with 10 µM of each compound. Untreated, staurosporine treated (STS, 10 μM) and DMSO treated (0.1% *v*/*v*) cells were used as control. Viability is indicated by color scale ranging from green (100% viability) to red (0% viability). Viability was calculated from DMSO control. (**B**) Measurement of caspase-3 activation after treatment with different natural compounds (10 µM) for 8 h. Fold induction was calculated by the DMSO control. (**C**) Compounds that were shown to be cytotoxic in our screening (compound code with corresponding name). Further names can be provided on request.

In order to identify a natural compound that might have an antineoplastic effect, 300 natural compounds (P01-P05, B-G, 02-11; Figure 2) were first examined during an initial screening process to determine whether cytotoxicity and apoptosis in the cell lines Jurkat J16 and Ramos could be observed (Figure 2). The screening was performed using MTT assay (Figure 2A) and caspase-3 assay (Figure 2B) with a final concentration of 10 µM of each compound per well. Cytotoxicity could be shown in several natural compounds (Figure 2C). Following literature research, three compounds of marine origin emerged as promising candidates for further research [4,6-dibromo-2-(2′,4′-dibromophenoxy)phenol (P01F03), 4,5,6-tribromo-2-(2′,4′-dibromophenoxy)phenol (P01F08), and 5-epi-nakijinone Q (P03F03)].

### 3.2. Cytotoxicity on Cell Lines Jurkat J16, Ramos, HL-60, THP-1, and Healthy PBMNCs

Initially, the compounds were further tested in Jurkat J16 and Ramos cells by performing repeated MTT assays as well as in the two acute myeloid leukemia cell lines HL-60 and THP-1. In addition to the anti-proliferative effect of the compounds on cancer cell lines, it was essential to investigate how the compounds affect healthy cells to identify a possible therapeutic window. The cells were incubated for 24 h or 72 h with eight different concentrations ranging from 0.01 µM to 30 µM of the respective compounds. As negative control, DMSO (0.1% *v*/*v*) was used. Staurosporine (10 µM), an indolocarbazole representing a strong inducer of apoptosis, was used as positive control. Following exposure, viability was determined using the MTT assay, while the corresponding IC50 values were calculated.

**Figure 3:**
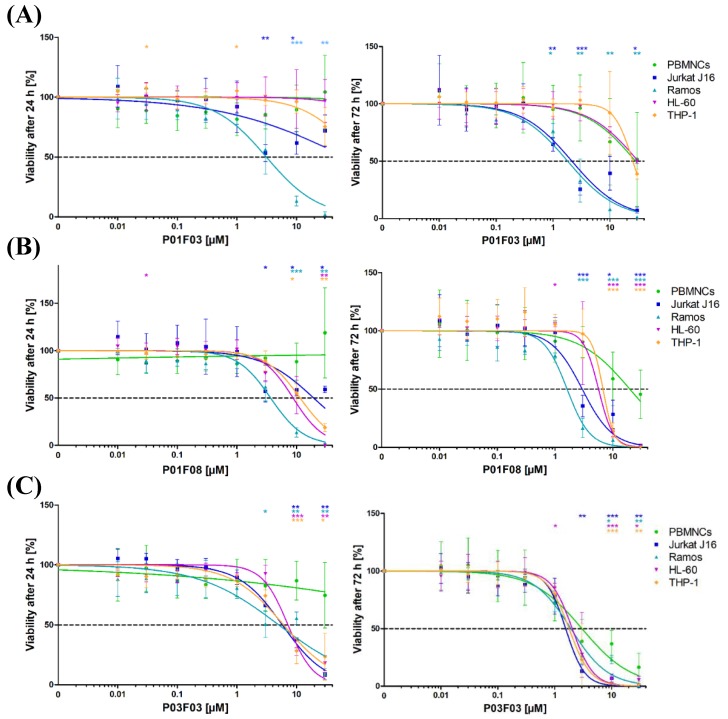
Cytotoxicity in cell lines Jurkat J16, Ramos, HL-60, and THP-1 and on healthy peripheral blood mononuclear cells (PBMNCs). MTT assay (viability) of Jurkat J16, Ramos, HL-60, and THP-1 and healthy PBMNCs after 24 h and 72 h of incubation with the compounds. (**A**) 4,6-dibromo-2-(2′,4′-dibromophenoxy)phenol (P01F03); (**B**) 4,5,6-tribromo-2-(2′,4′-dibromophenoxy)phenol (P01F08); and (**C**) 5-epi-Nakijinone Q (P03F03). The data are shown as mean ± SD of three individual experiments in cell lines (Jurkat J16, Ramos, HL-60, THP-1), five individual experiments in PBMNCs after 24 h incubation, and nine individual experiments in PBMNCs after 72 h incubation. All experiments were performed in triplicates. The values are normalized to staurosporine (STS, 10 μM) and DMSO (0.1% *v*/*v*). Welch’s t-test was used to detect statistically significant differences between PBMNCs and cell lines. Statistical significance was established at asterisks displaying *p*-values * *p* < 0.05, ** *p* < 0.01, *** *p* < 0.001.

We observed a clear time and concentration dependent decline in viability for all three compounds. P01F03 did not cause sufficient cytotoxicity in PBMNCs, Jurkat J16 cells, HL-60 cells, and THP-1 cells to obtain an IC50 value after 24 h incubation. After 72 h incubation, viability of all cells decreased with IC50 values ranging from 1.81 µM to 31.4 µM (Figure 3A) with significant differences between PBMNCs and the lymphatic cell lines.

After incubation with P01F08 for 24 h, only the PBMNCs were hardly effected. IC50 values for all cell lines ranged from 3.68 to 19.38 µM. After 72 h incubation with P01F08, the PBMNCs reached an IC50 value of 19.62 µM while the cell lines ranged from 1.61 to 6.74 µM (Figure 3B). In the higher concentrations of 3, 10, and 30 µM, a significant difference between the effect on PBMNCs and all cell lines was observed.

P03F03 showed overall higher cytotoxic effects. IC50 values ranging from 5.04 to 7.18 µM were found for the cell lines, while PBMNCs viability did not decline under 50% after 24 h incubation. In contrast, all cell viability was strongly affected after 72 h incubation with P03F03. IC50 values ranged from 1.56 to 3.01 µM (Figure 3C). Because of the equally strong effect of P03F03 on the healthy PBMNCs, a greater therapeutical window for this compound was not expected. The exact IC50 values with the corresponding 95% confidence interval are shown in Table 2.

### 3.3. Induction of Apoptosis in Ramos and Jurkat J16 Cells in Comparison to Healthy PBMNCs

In addition, cytotoxicity was assessed by the PI uptake assay using culture medium and DMSO as negative controls, while staurosporine (STS, 10 µM) was used as positive control [14]. This is a measurement in which the proportion of dead cells can be evaluated. In principle, this method cannot discriminate between late apoptosis and necrosis. However, in order to measure apoptotic activity, the cells were incubated with and without the multi caspase inhibitor QVD [15]. As a result, a statement about the potential induction of apoptosis could be made. The PI uptake was measured for the cell lines Jurkat J16 and Ramos with the two incubation times, 24 h and 72 h.

**Figure 4:**
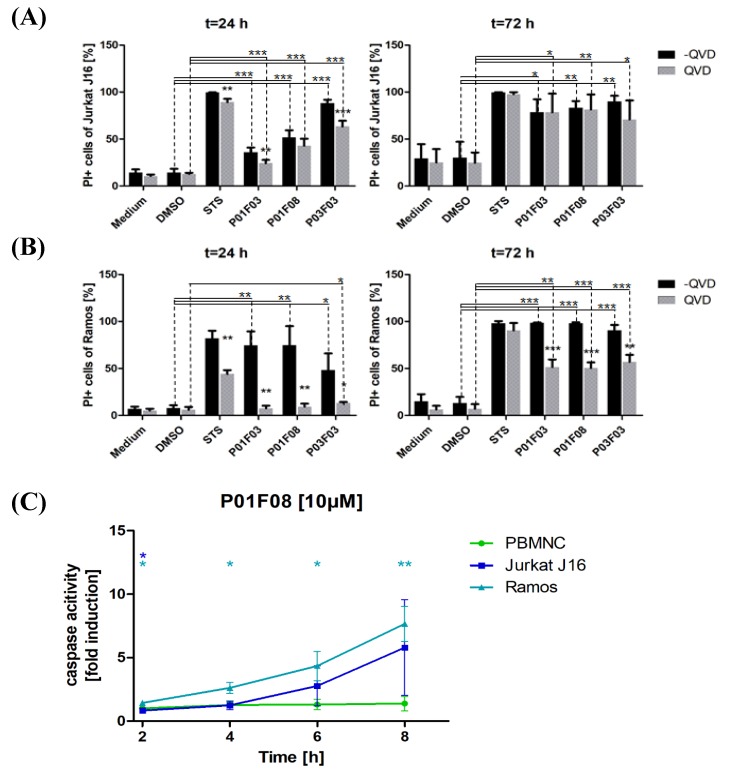
Propidium iodide (PI) uptake in the cell lines (**A**) Jurkat J16 and (**B**) Ramos with and without caspase inhibitor Q-VD-OPh (QVD) with 10 µM of each compound and incubation times of 24 h and 72 h. The results are shown as mean ± SD of three independent experiments, which were performed in triplicates. Welch’s t-test was used to detect statistically significant differences. Significance between treated and untreated groups is indicated with asterisks above the columns. Significance between DMSO and different cells is shown with asterisks on lines above displaying *p*-values * *p* < 0.05, ** *p* < 0.01, *** *p* < 0.001. (**C**) Kinetics of caspase-3 activity in PBMNCs and in cell lines Jurkat J16 and Ramos after the stimulation for up to 8 h with 4,5,6-tribromo-2-(2′,4′-dibromophenoxy)phenol (P01F08). The results are shown as mean ± SD of triplicates. The values are normalized to DMSO. Welch’s t-test was used to detect statistically significant differences between PBMNCs and cell lines and are established at asterisks displaying *p*-values * *p* < 0.05, ** *p* < 0.01, *** *p* < 0.001.

After 24 h of incubation, a difference in the PI uptake of the cells exposed to the compounds only and those co-incubated with QVD could be noticed. In Jurkat J16 cells, this effect was less pronounced than in Ramos cells (Figure 4A,B). There was a difference in PI uptake in cells exposed to the natural compounds without QVD in P01F08 and a significant difference in P01F03 and P03F03 after 24 h of incubation. These findings indicated that the compounds acted, at least partially, via the induction of apoptosis.

After 24 h of incubation, a clear and significant difference between the samples P01F03 and P01F03 + QVD (*p* = 0.0079) and P03F03 and P03F03 + QVD (*p* < 0.0004) could be observed. 

After 72 h, a significant difference between the DMSO control and the treated groups could be obtained. However, at this time, 78.4% of the Jurkat J16 cells treated with P01F03 incorporated PI, irrespective of the presence of QVD (also 78.4%). Similarly, Jurkat J16 cells incubated with P01F08 reached a PI uptake of 83.4% without QVD and 81.5% with QVD (Figure 4A). In Ramos cells (Figure 4B), the PI uptake showed a greater difference in the presence of QVD after 72 h. Without QVD, 98.6% of Ramos cells treated with P01F03 incorporated PI; in the presence of QVD, 51.5% of the cells incorporated PI. For P01F08, the PI uptake sunk from 98.2% without QVD to 50.4% with QVD. For P03F03, the difference was slightly smaller (90.6% PI uptake without QVD, 57% PI uptake with QVD). All compounds showed a significant difference in co-exposure with QVD (P01F03: *p* = 0.0005; P01F08: *p* = 0.0002; P03F03: *p* = 0.0035).

The high degree of PI uptake following the exposure of the cells to the compounds confirmed the cytotoxicity of the compounds and was in line with the results of the MTT assay. Furthermore, the co-exposure with QVD in our preliminary experiments gave rise to the hypothesis that the compounds acted via the induction of apoptosis. To confirm the apoptosis inducing ability of P01F08, which turned out to have a therapeutic gap (Section 3.2), we used the DEVDase assay for measurement of the caspase-3 activity as an indicator for apoptosis. 

In preliminary experiments with staurosporine, strong apoptosis was induced in all cell types (data not shown). Following exposure of Jurkat J16 and Ramos cells (Figure 4C) to P01F08 in three independent experiments, a rise of caspase-3 activity could be observed. The increase was not as high as observed in the cells exposed to STS. Still, P01F08 exerted a caspase-3 activity, which increased as a function of the time of exposure (2 h vs. 8 h: Ramos *p* = 0.0154; Jurkat *p* = 0.15). The caspase-3 activity of P01F08 was also determined for the PBMNCs by the DEVDase assay. Following exposure to P01F08, there was no stringent increase in caspase-3 activity compared to the malignant counterparts (Figure 4C). In line with the preliminary results (Section 3.2), no significant caspase activity in PBMNCs was observed, even after eight hours of testing. The different effects were particularly clear for Ramos, and significant differences between Ramos cells and healthy PBMNCs at all times could be observed (2 h: *p* = 0.0112; 4 h: *p* = 0.0161; 6 h: *p* = 0.0354; 8 h: *p* = 0.0091) while for Jurkat cells, a statistically significant difference could only be observed after 2 h (*p* = 0.038) (Figure 4C). 

### 3.4. Differentiation Capacity of Healthy PBMNCs Following Exposure to P01F08

**Figure 5:**
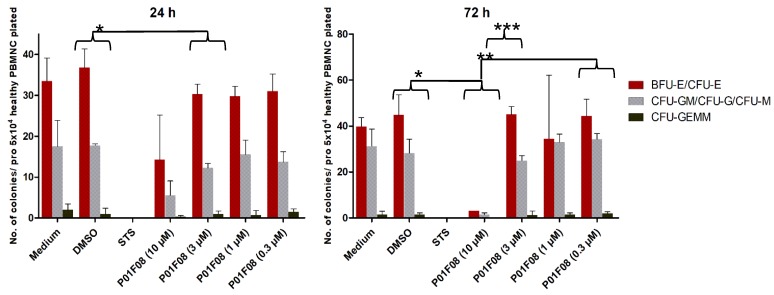
Colony forming units (CFU) assay with PBMNCs after 24 h and 72 h of treatment with 4,5,6-tribromo-2-(2′,4′-dibromophenoxy)phenol (P01F08) in different concentrations. The bars represent the mean ± SD of two patient samples performed in duplicates. Colonies were differentiated in red precursors (colony-forming unit-erythroid, CFU-E; burst-forming unit-erythroid, BFU-E), white precursors (colony-forming unit-granulocyte, -monocyte, CFU-GM; colony-forming unit-granulocyte; CFU-G; colony-forming unit-monocyte, CFU-M), and colony-forming unit-granulocyte, -erythrocyte, -monocyte, -megakaryocyte (CFU-GEMM) by light microscopy. Welch’s t-test was used to detect statistically significant differences between all colonies of DMSO and the different concentration of P01F08. Statistical significance was established at asterisks display *p*-values * *p* < 0.05, ** *p* < 0.01, *** *p* < 0.001, **** *p* < 0.0001.

In our preliminary results, P01F08 stood out as a promising apoptosis inducing compound that might open a therapeutic window. In a next step, PBMNCs enriched for HSPCs were incubated with compound P01F08 for 24 h and 72 h before they were plated in a CFU assay to investigate the proliferation and the differentiation of clonogenic progenitor cells (red precursor, white precursor, CFU-GEMM). For both incubation times, a clear decrease in the number of colony-forming cells was observed at a concentration of 10 μM. In fact, the cells that were incubated for 72 h with 10 µM of the compound showed a reduction of 94.0% of total colonies compared to the DMSO control (number of total colonies DMSO 74.5 vs. P01F08 10 µM 4.5; *p* = 0.0199) and the other concentration (3 µM: *p* < 0.0001; 0.3 µM: *p* = 0.0016). At concentrations of 0.3, 1, and 3 μM, the proliferation and the differentiation were hardly affected, independent of the incubation time (Figure 5).

### 3.5. Cytotoxicity on Primary Malignant Cells Obtained from Patients with AML

With proven efficacy on myeloid cell lines (Section 3.2), we were interested in the effect of the compounds on primary patient cells. Blood samples were obtained with written informed consent of six patients with an untreated AML and blast excess in the peripheral blood (Table 1). The mononuclear cells were obtained using a density gradient centrifugation. For determination of viability, MTT assays were performed. 

**Figure 6:**
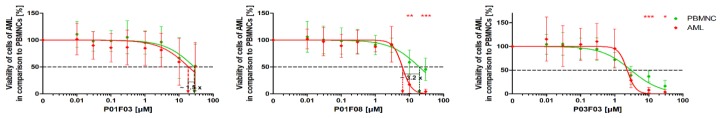
Cytotoxicity on primary malignant cells obtained from patients with AML. MTT assays (viability) of cells from patients with acute myeloid leukemia (*n* = 6) in comparison to cells from healthy donors (*n* = 9), as previously shown in Figure 3, after 72 h of incubation with compounds 4,6-dibromo-2-(2′,4′-dibromophenoxy)phenol (P01F03), 4,5,6-tribromo-2-(2′,4′-dibromophenoxy)phenol (P01F08), and 5-epi-Nakijinone Q (P03F03). The results are shown as mean ± SD of the experiments performed in triplicates. The values are normalized to staurosporine (STS, 10 μM) and DMSO (0.1% *v*/*v*). Welch’s t-test was used to detect statistically significant differences between healthy PBMNCs and AML cells. Statistical significance was established at asterisks display *p*-values * *p* < 0.05, ** *p* < 0.01, *** *p* < 0.001.

Following incubation of AML patients’ cells with P01F03, an IC50 value of 19.22 µM (9.36–39.44 µM) was reached. The IC50 value of P01F08 on primary malignant cells amounted to 6.22 µM (4.55–8.50 µM), and P03F03 showed an IC50 value of 2.32 µM (1.47–3.67 µM). Comparing the IC50 values of the healthy PBMNCs with the IC50 values of the AML patients, P01F03 showed a difference of 8.93 µM. This corresponded to 1.5-fold lower toxicity in healthy PBMNC compared to primary AML cells. However, there was no statistical significance. P01F08 exhibited a difference of 13.4 µM and therefore corresponded to a 3.2-fold higher affection of primary malignant cells compared to healthy counterparts. At higher concentrations of 10 µM and 30 µM, P01F08 and P03F03 showed clear and significant differences in cytotoxicity between primary AML cells and the healthy counterparts (P01F08: 10 µM: *p* = 0.0014, 30 µM: *p* = 0.0003; P03F03: 10 µM: *p* < 0.0001, 30 µM: *p* = 0.0196). However, the IC50 value of P03F03 only differed by 0.7 µM in healthy PBMNCs to the cytotoxicity in AML cells (Figure 6).

## 4. Discussion

Following an extensive screening of 300 natural compounds contained within a natural product library established at the Institute of Pharmaceutical Biology and Biotechnology, Heinrich Heine University Düsseldorf, we focused on three candidate compounds because they had a clear-cut, dose-dependent antineoplastic effect on two model cell lines, Jurkat (T-cell) and Ramos (B-cell). In further tests, the IC50 values of the compounds for Jurkat J16 and Ramos cells following 72 h of incubation varied between 1.61 and 2.95 μM. Jurkat J16 cells had a higher proliferated rate than Ramos cells. According to the German Collection of Microorganisms and Cell Culture, the doubling time for Ramos cells is about 48 h [16] and for Jurkat about 25–35 h [17]. Ramos cells were more affected than Jurkat cells after 24 h. This could be an indicator for the underlying mechanism. Potentially, the compounds need to be enriched in the cells before apoptosis is induced. In cells with lower proliferation rates, this would lead to a higher cytotoxicity after a short period of time. It is interesting to note that, unlike other cytotoxic drugs, the effect of the compounds after 24 h did not inevitably depend on the cumulative population doubling (CPD). In the used cell lines, the effect was more pronounced in slower proliferating cell lines (Jurkat J16 and THP-1) after 24 h. This suggested a cytostatic effect independent of the cell division rate.

Compared to the IC50 values of Jurkat J16 and Ramos cells, the IC50 values observed for PBMNCs obtained from healthy donors were significantly higher. In other words, while the cytotoxicity in the cell lines already reached 50%, normal PBMNCs were unaffected. These findings implied that normal blood cells were less susceptible to the toxic effects of the compounds. 

When investigating apoptosis induction by PI uptake with the usage of QVD, a clear trend could be shown. As QVD is an apoptosis inhibitor, cells that die from necrosis would have a higher intake in PI than cells that die apoptotic. The significant differences between the PI uptake in Jurkat J16 and Ramos cells that were treated with or without QVD suggested that the compounds induced apoptosis. It was noticeable that the compounds had a lower effect on Jurkat J16 cells than on Ramos cells after 24 h of incubation. When comparing the results from the MTT assays and the results of PI uptake, a relationship was recognizable. For example, after 24 h of incubation with 10 µM P01F03, about 38% of the Jurkat J16 cells died according to the MTT assay. The PI uptake also showed 36% of PI uptake in cells without QVD. The differences between Jurkat and Ramos cells could therefore be attributed to the reasons mentioned above. Keeping in mind that the compounds might have needed to be enriched first, the incubation time of 24 h was not long enough for the compounds to accumulate in the cells in a way that apoptosis was induced. The lower CPD of Ramos cells might have enabled the compounds to enrich in one cell enough for apoptosis induction. After 72 h of incubation, the gap between cells treated with QVD and cells without co-incubation with QVD closed. Because of the longer incubation time, the cells may have undergone apoptosis despite the protective effects of QVD and reached the stage of secondary necrosis.

Because the difference in induced cytotoxicity between PBMNCs and lymphatic and myeloid cell lines was highest for P01F08, this compound was analyzed in further tests. The results of the PI uptake were supported by a DEVDase assay showing that the lymphatic cell lines Jurkat J16 and Ramos had higher caspase-3 activity than healthy PBMNCs. Keeping in mind that the healthy PBMNCs showed no decrease in viability in the MTT assays after 24 h, the results of the DEVDase assay can be explained. There was hardly any caspase-3 activity, even after 8 h of incubation with P01F08. This might have been due to the lack of cell toxicity after a short incubation time. 

Moreover, the results of our colony-forming unit assays showed that even the sensitive hematopoietic progenitor cells were not significantly affected in their growth by P01F08 at a concentration of 3 μM. This difference could be relevant, as it reflects a potential therapeutic window permitting the use of these substances for the development of antineoplastic drugs for the treatment of patients with hematological malignancies. We also included the myeloid derived cell lines HL-60 and THP-1 in our analysis and found that the results obtained were at least partially similar to those observed with the T- and the B-cell derived cell lines. Considering the potential pitfalls associated with the use of cell lines kept in culture for a long time, we extended our experiments using freshly prepared leukemic cells from patients with acute myeloid leukemia. Based on the samples of six different and newly diagnosed patients with AML, prior to therapy, we examined their leukemic blasts excess in the peripheral blood. Compared to the IC50 values of healthy donors, we found that the IC50 values were even smaller, thereby pronouncing the gap between normal and malignant cells. In particular, P01F08 showed a 3.2-fold lower IC50 value in primary leukemic cells (6.22 µM; 4.55–8.50 µM) compared to the PBMNC of healthy donors (19.62 µM; 13.28–28.98 µM). Considering the fact that primary malignant cells behave substantially differently from most cell lines, our findings demonstrate, for the first time, the therapeutic relevance of these compounds for patients with AML [18]. Differently from us, Li et al. investigated the effect of cytostatic agents on mesenchymal stem cells, on healthy PBMNCs, and on the acute promyelocytic leukemic cell line NB-4. For the cytostatic drug Paclitaxel, there were significantly higher differences between the leukemic cell line (IC50: 0.223 × 10^−7^ mol/L, ±0.001) and the healthy PBMNCs (IC50: 56.2 × 10^−7^ mol/L, ±0.8). Of note, paclitaxel is rarely used in patients with hematological malignancies for whom cytarabine and cyclophosphamide are major components within most of the treatment protocols. These two drugs show similar IC50 values compared to P01F08 (Table 3) [19]. 

Strese et al. conducted a study comparing the effects of Melflufen and Melphalan on different leukemic cell lines, including primary AML cells, with the effects on healthy PBMNCs. Melflufen showed a 7.5-fold higher IC50 value on healthy PBMNCs compared to primary AML cells, whereas Melphalan showed a 1.8-fold higher IC50 value on PBMNCs. In contrast to Melflufen, Melphalan is a classical alkylating cytostatic drug that is used in hematological malignancies as a conditioning regimen before blood stem cell transplantation or at a lower dose with palliative intention. Therefore, Melphalan is well suited to compare its IC50 values with those of P01F08 (Table 2) [20], keeping in mind that different cytotoxicity tests were used in the two studies {MTT vs. FMCA (fluorometric microculture cytotoxicity assay), Strese et al. vs. XTT 2,3-bis(2-methoxy-4-nitro-5-sulphophenyl)-5-[(phenylamino) carbonyl]-2H-tetrazolium hydroxide, Li et al.}.

There are little data on some derivatives of the polybrominated diphenyl ethers. For instance, PANC-1 cells were exposed to the derivates 3,4,5-tribromo-2-(2′,4′-dibromophenoxy)-phenol and 3,5-dibromo-2-(2′,4′-dibromophenoxy)-phenol, respectively. Under glucose-starved conditions, IC50 values of 2.1 and 3.8 μM were observed. Using similar culture conditions, no anti-proliferative activity was observed up to a concentration of 30 μM. In the search for the mechanisms underlying the anti-proliferative effect, the authors assumed that 3,4,5-tribromo-2-(2′,4′-dibromophenoxy)-phenol inhibited complex II in the mitochondrial electron transport chain [21] potentially favoring apoptosis. This view would be in line with our findings showing apoptosis as a prominent mechanism for the toxic effect observed in our cells independent of their origin. The story is different as far as P03F03 is concerned. It can be isolated from the sponge *Dactylospongia metachromia,* which is a part of the genus *Dactylospongia,* a source of bioactive secondary metabolites. These compounds are interesting, as their biological activities include antitumor, anti-inflammatory, and antiviral activities. The species was originally described as *Hippospongia metachromia* by De Laubenfels in 1954 [13]. Using the MTT assay with L5178Y mouse lymphoma cells as the target, P03F03 was cytotoxic with an IC50 value of 1.1 μM. When tested against 16 protein kinases involved in the regulation of tumor growth and metastasis, P03F03 inhibited anaplastic lymphoma kinase (ALK), focal adhesion kinase (FAK), insulin like growth factor (IGF11-R), proto-oncogene SRC, and vascular endothelial growth factor (VEGF-R2 R2) [13]. Other compounds with a nakijiquinone core structure, such as P03F03, have been isolated from *Spongia* sp., *Dactylospongia elegans*, *Smenospongia* sp., and *Hippospongia* sp. These compounds have received great attention, as they have a wide range of biological activities, such as cytotoxic, anti-microbial, and inhibitory activity against the tyrosine kinase epidermal growth factor receptor (EGFR) and protein kinase C [13]. Similar to the published results, our findings confirm the anti-neoplastic effect of the compound on Jurkat J16 and Ramos cell lines via the induction of apoptosis. Still, we found that this compound was also cytotoxic for healthy PBMNCs, as reflected by the similar IC50 values. In our view, this natural compound in its present form without structural modifications is not suitable for further drug development. 

Based on a large library of natural compounds with antineoplastic activity, we propose a stepwise approach using a variety of different methods to assess the therapeutic potential of candidate compounds. Particular emphasis is laid on the use of primary human cells—both normal and malignant—to come as close as possible to the true in vivo situation, avoiding inherent artifacts related to the use of cell lines.

## Figures and Tables

**Table 1:** Patient demographics and clinical characteristics.

Characteristic	No.	%
No.	6	
Median age, years	67	
Range	40–74	
*Sex*		
Male	2	33.3
Female	4	66.7
*AML*		
WHO		
With recurrent genetic abnormalities	4	66.7
MDS-related changes	2	33.3
*Karyotype*		
Normal	5	84.3
Aberrant	1	16.7
*Molecular/genetic risk*		
Favorable	3	50
Intermediate	1	16.7
Adverse	2	33.3
Median blasts BM, %	75	
Range	50–99	
Median ANC, ×10^9^/L	2.119	
Range	0.189–5.248	
Median Hb, g/dL	8.2	
Range	4.3–9.6	
Median plts, ×10^9^/L	59.0	
Range	12.0–168.0	
Median blasts PB, %	54	
Range	18–92	

Abbreviations: AML, acute myeloid leukemia; ANC, absolute neutrophil count; BM, bone marrow; Hb, haemoglobin; MDS, myelodysplastic syndrome; No., number; NOS, not otherwise specified; PB, peripheral blood; WHO, World Health Organization. Molecular and genetic risk was classified according to the European Leukemia Net (ELN) 2017 criteria.

**Table 2:** Mean IC50 values (with 95% confidence intervals) of Melphalan in four different cell lines and AML patient samples and PBMNCs from healthy donors compared to the compounds P01F03, P01F08, and P03F03 (Melphalan values are from Strese et al., 2017).

Cells	Melphalan IC50 (µM)	P01F03 IC50 (µM)	P01F08 IC50 (µM)	P03F03 IC50 (µM)
MV4-11	1.7 (1.4–2.0)			
HL-60	6.1 (5.5–6.9)	31.4 (21.38–46.11)	5.65 (4.25–7.52)	2.03 (1.54–2.69)
Kasumi	3.8 (3.3–4.5)			
KG1-A	8.6 (7.1–10)			
THP-1		25.26 (19.92–32.03)	6.74 (3.88–11.71)	1.79 (1.44–2.22)
Jurkat J16		2.15 (1.267–3.630)	2.95 (2.08–4.18)	1.56 (1.27–1.91)
Ramos		1.81 (1.35–2.42)	1.61 (1.30–1.99)	1.92 (1.55–2.39)
Patients	5.5 (4.7–6.5)	19.22 (9.36–39.44)	6.22 (4.55–8.50)	2.32 (1.47–3.67)
PBMNC	9.7 (5.0–19)	28.15 (16.58–47.78)	19.62 (13.28–28.98)	3.01 (2.18–4.16)

**Table 3:** Chemosensitivity of NB-4 cells and PBMNCs [IC50(10)^7^ mol/L]. The results represent the mean ± SEM of triplicate cultures of one representative experiment. NB-4 cell, acute promyelocytic leukemia cell line (adapted from Li et al., 2004).

Agent	NB-4 Cell	PBMNC	Factor (PBMNC/NB-4 Cell)
Cytarabine	29.2 ± 3.9	46.3 ± 7.4	1.58
Busulphan	390 ± 28	154 ± 43	0.39
Cyclophosphamide	4900 ± 210	12,600 ± 800	2.57
